# Depression in Romanian Medical Students—A Study, Systematic Review, and Meta-Analysis

**DOI:** 10.3390/jcm14165853

**Published:** 2025-08-19

**Authors:** Corina Lavinia Duica, Silvius Ioan Negoita, Alina Pleșea-Condratovici, Lavinia-Alexandra Moroianu, Mariana Daniela Ignat, Pantelie Nicolcescu, Anamaria Ciubara, Karina Robles-Rivera, Liliana Mititelu-Tartau, Catalin Pleșea-Condratovici

**Affiliations:** 1 Psychiatry Department, Faculty of Medicine, Lucian Blaga University, 550169 Sibiu, Romania; lavinia.duica@ulbsibiu.ro; 2 Faculty of Medicine, University of Medicine and Pharmacy Carol Davila, 020021 Bucharest, Romania; silvius.negoita@umfcd.ro; 3 Faculty of Medicine and Pharmacy, “Dunarea de Jos” University, 800008 Galati, Romania; mariana_daniela52@yahoo.com (M.D.I.); pantelie.nicolcescu@ugal.ro (P.N.); anamaria.ciubara@ugal.ro (A.C.); catalin.plesea@ugal.ro (C.P.-C.); 4 Faculty of Medicine, National Autonomous University of Mexico (UNAM), Mexico City 04510, Mexico; krobles@facmed.unam.mx; 5 Department of Pharmacology, Faculty of Medicine, “Grigore T. Popa” University of Medicine and Pharmacy, 700115 Iasi, Romania; lylytartau@yahoo.com

**Keywords:** depression, students, medical, mental health, personality, prevalence, psychiatric status rating scales, time management, Europe, eastern

## Abstract

**Background:** Depression is a significant global mental health concern, especially among medical students. This study combines two components: (1) a cross-sectional assessment of depression and related psychological and demographic factors among students at “Dunărea de Jos” University of Galați, and (2) a systematic review and meta-analysis of published Romanian studies on depression in medical students. **Methods:** For the cross-sectional component, 495 students (Years I–III) completed online questionnaires assessing depressive symptoms (PHQ-9), personality traits, procrastination, and sociodemographic factors. In the systematic review, studies from PubMed and Web of Science were synthesized following PRISMA guidelines, with prevalence data being pooled via random-effects meta-analysis. **Results:** In the Galați sample, 34.0% of students had clinically significant depressive symptoms (PHQ-9 ≥ 10). Depression was associated with female gender, being in the third year of study, low social support, high neuroticism, and procrastination. The meta-analysis (six studies, N = 1546) yielded a pooled national prevalence of 19.99% (95% CI: 18.24–21.73%). **Conclusions:** Depression is highly prevalent among Romanian medical students, particularly in Galați. The findings support the need for targeted mental health interventions in Romanian universities. Registration: This systematic review has been registered in the Prospero database (registration number CRD420251056873).

## 1. Introduction

Depression is a leading global cause of disability, exerting a profound burden on individuals, families, and healthcare systems [1,2]. As a widespread public health concern, it affects hundreds of millions of people worldwide [1]. While the prevalence rates vary by country and demographic group, European epidemiological studies consistently report high rates of depression [3]. In Romania, although comprehensive national data remain limited and methodologically inconsistent [4,5], the available evidence suggests a substantial impact of depression on the population [6,7].

University students, particularly those in medicine and health sciences, show significantly higher levels of psychological distress compared to their non-student peers [8]. Medical students in particular are disproportionately affected by anxiety, depression, and suicidal ideation [9,10,11], with international meta-analyses estimating that 25–33% screen positive for depressive symptoms—substantially higher than general population rates [9,12]. This increased vulnerability is linked to key stressors within medical training environments, including academic overload, competition, financial pressure, exposure to patient suffering, sleep deprivation, and work–life imbalance [13,14,15]. Romanian studies echo these findings, reporting high rates of depression, anxiety, and stress among students in medical, dental, and nursing programs [16,17,18,19,20,21].

This study also includes a systematic review and meta-analysis of the prevalence of depression among Romanian medical and dental students in order to better synthesize the currently available national evidence and to provide a solid framework for new regional data.

In spite of this body of work, knowledge on student depression in certain areas of Romania remains limited. Furthermore, relatively little research has been done on how depression interacts with specific psychological traits, such as procrastination, in Romanian medical students. Moreover, no previous study has attempted to directly integrate institution-level data with national estimates through a combined approach.

Methodological inconsistencies, such as varying assessment timelines in relation to academic stressors (like exam periods) and publication in non-peer-reviewed journals, make obtaining comprehensive knowledge even more difficult. To address this critical gap, our study offers a novel dual approach: (1) providing new cross-sectional data on the prevalence and correlates of depression among medical and nursing students at the “Dunărea de Jos” University of Galați, and (2) conducting the first systematic review and meta-analysis of the prevalence of depression among Romanian medical students. This integration of local and national data offers a more robust and contextually grounded understanding of mental health challenges in Romanian medical education.

Depression, procrastination, and personality traits, particularly neuroticism, conscientiousness, and extraversion, are central constructs in understanding psychological vulnerability among medical students. Depression is commonly defined as a mood disorder characterized by persistent sadness, loss of interest, and cognitive impairments such as poor concentration and feelings of worthlessness. In the academic context, procrastination is conceptualized as the intentional delay of important tasks despite foreseeable negative consequences, and is increasingly viewed as a maladaptive coping strategy linked to stress, guilt, and psychological distress. From a personality perspective, the Five-Factor Model (FFM) identifies neuroticism as the predisposition to experience negative emotions and stress sensitivity; it identifies conscientiousness as reflecting self-discipline, organization, and goal-directed behavior; and it associates extraversion with sociability and emotional expressiveness. Prior international studies and meta-analyses have consistently found that higher neuroticism and procrastination, and lower conscientiousness and extraversion, are linked to increased depressive symptoms. These factors are hypothesized to interact within the cognitive-behavioral framework of depression, wherein emotional dysregulation, avoidant behaviors, and diminished executive function contribute to vulnerability. By evaluating these variables in Romanian medical students, our study aims to test these well-established associations in a context with limited prior empirical data.

Complementing this local investigation, a systematic review and a meta-analysis of existing Romanian studies were conducted to situate our findings within a national framework. Based on previous research and the observed local factors, we tested the following hypotheses within the study sample: (1) a high prevalence of clinically significant depression; (2) higher reported levels of depressive symptoms among female students and third-year students compared to their counterparts; (3) significant positive associations between depression scores and psychological traits including high neuroticism, high procrastination, low conscientiousness, and low extraversion; and (4) a significant association between higher depression scores and contextual factors like low social support and cumulative risk exposure.

Despite evidence of elevated depression rates among Romanian medical students, the existing studies are limited in number, often geographically restricted, and methodologically heterogeneous. No prior research has combined institutional-level data with a national-level synthesis. In light of these gaps, the present study had two main objectives. First, it aimed to assess the prevalence and psychological correlates of depression among medical and nursing students at “Dunărea de Jos” University of Galați, focusing on personality traits, procrastination, sociodemographic factors, and contextual stressors. Second, it sought to synthesize the existing national data through a systematic review and meta-analysis of published studies on the prevalence of depression among Romanian medical, dental, and nursing students. By combining institutional-level data with national-level estimates, this study aims to offer a more comprehensive understanding of student mental health in Romania and inform future preventive and support strategies in medical education. It complements international findings by incorporating psychological predictors that are not commonly included in existing meta-analyses, such as procrastination and personality traits.

## 2. Materials and Methods

### 2.1. The Cross-Selectional Study

This study used a quantitative, cross-sectional, observational approach. We used correlational and descriptive statistics to assess the relationships between procrastination, depression, and a number of personal, psychosocial, and demographic factors in the students sample.

#### 2.1.1. Participants and Procedure

The study population consisted of undergraduate students enrolled in the medical and nursing programs at the “Dunărea de Jos” University of Galaţi, Romania, for the 2024–2025 academic year. Participants were chosen from the first, second, and third years of study. The focus on years I–III was intentional, as these represent the preclinical and early clinical phases of training, a period during which students are often adjusting to the intense demands of medical education. These years are typically associated with elevated stress, identity shifts, and the development of coping mechanisms. Limiting the sample to this subset allowed for a more homogeneous group in terms of academic exposure, while avoiding confounding factors present in more advanced students, such as final-year exam pressure, licensing preparation, and greater clinical responsibility. This approach aligns with prior research that identifies early medical training years as a high-risk window for mental health deterioration.

The sample of 495 students represents a substantial portion (approximately 40%) of the total Romanian medical and nursing students enrolled in these first three years at the university, providing a statistically significant sample size that is sufficient for estimating prevalence and exploring correlates within this population. The inclusion of students from years I–III also allows for potential comparisons across different stages of the curriculum, as this range encompasses both preclinical and the initial clinical exposure phases. The sample was not stratified but included a naturalistic distribution of students from all three years. No exclusion criteria were applied apart from enrollment status. Recruitment was conducted via various channels: in-classroom announcements by instructors; WhatsApp messages distributed through student group chats; emails sent via university mailing lists.

Inclusion criteria were as follows: active enrollment in either the medical or nursing program; first-, second-, or third-year student status during the mentioned time interval; informed consent provided electronically prior to participation. Participation was entirely voluntary. Students received no incentives, and participation had no bearing on academic performance or evaluations. Before starting the survey, participants reviewed an online informed consent form detailing the following: the study’s purpose and procedures; data anonymity and confidentiality; their right to withdraw at any time without penalty. Only students who clicked “I agree” on the electronic consent form could access the questionnaire. The survey was designed to take approximately 30–40 min. Individual data were coded anonymously using identification numbers for secure storage and analysis. To reduce selection bias, participation was fully voluntary and unrelated to academic grading or instructor involvement.

#### 2.1.2. Measures

To assess depressive symptoms, procrastination, and personality traits, we selected widely used, validated psychometric instruments that are suitable for online administration and have strong international psychometric properties. Tools were selected based on their relevance to our hypotheses, prior use in student mental health studies, and suitability for rapid digital deployment through the PsyToolkit platform version: 3.6.2 [22]. The examination consisted of the following:

- Context and socio-demographic data: A customized questionnaire was used to gather data like age, gender, year of study, faculty, marital status, living situation, parental education, exposure to negative behavioral models during childhood and adolescence, presence of hobbies, and social support;
- Depression: The Patient Health Questionnaire-9 (PHQ-9) [23] was used to assess the severity of depressive symptoms over the past two weeks. The PHQ-9 is a 9-item self-report measure scored on a 4-point Likert scale (0 = Not at all, 3 = Nearly every day), yielding a total score from 0 to 27. Example item: “Feeling down, depressed, or hopeless”. Standard cut-offs were used for severity categories: 0–4 (minimal), 5–9 (mild), 10–14 (moderate), 15–19 (moderately severe), 20–27 (severe). In our sample, the Cronbach’s alpha was 0.88, indicating high internal consistency;
- Procrastination was measured using the General Procrastination Scale—Student Version [24], a 20-item self-report instrument that assesses tendencies toward task delay and academic procrastination. Participants rated items on a 5-point Likert scale (from 1 = “Extremely uncharacteristic of me” to 5 = “Extremely characteristic of me”). Sample item: “I often find myself performing tasks that I had intended to do days before.” Several items are reverse-coded. This instrument has demonstrated good reliability in academic populations; in our sample, the Cronbach’s alpha was 0.85;
- Personality traits: We assessed personality using the Big Five Inventory—Short Form (BFI-S) [25], which includes 15 items that measure five traits: neuroticism, extraversion, openness, agreeableness, and conscientiousness. Each trait is measured by 3 items, with responses being given on a 7-point Likert scale (from 1 = “Strongly disagree” to 7 = “Strongly agree”). Sample neuroticism item: *“I see myself as someone who gets nervous easily.”* In our sample, the subscale reliability (Cronbach’s alpha) ranged from 0.68 (agreeableness) to 0.81 (neuroticism), consistent with validation studies of the short-form BFI.

Responses on contextual risk exposure (e.g., early exposure to antisocial models) were captured using four dichotomous items (Yes/No) and recoded into thematic binary or cumulative variables.

#### 2.1.3. Statistical Analysis

##### Data Preparation and Screening

Raw data exported from PsyToolkit were managed and prepared using Microsoft Excel, which involved aggregation, cleaning, and variable recoding. Responses from items utilizing a checkbox format or multiple dichotomous variables were systematically transformed into more interpretable categorical or composite variables. Where appropriate, this involved creating synthetic variables through the logical clustering or summation of binary combinations based on theoretical relevance.

Exposure to negative behavioral models: Exposure to antisocial or negative behaviors (e.g., rule-breaking, animal cruelty, illegal activities, parental conviction), assessed via four binary items, was recoded into (1) a general binary variable exposed_antisocial (0 = no, 1 = yes if any item checked); (2) specific thematic binary variables: exposed_rules (1 if modelsbad_1 = 1), exposed_animal_violence (1 if modelsbad_2 = 1), exposed_illegality (1 if modelsbad_3 = 1), exposed_conviction (1 if modelsbad_4 = 1). For correlational analyses assessing cumulative exposure, a score was calculated by summing the ‘yes’ responses across the four items (range 0–4).

Social support: The variable measuring perceived social support was transformed into an ordinal variable with three levels: 0 = No support, 1 = Minimal support (one source), 2 = Extended support (multiple sources).

Parental education: Parental education levels, collected via checkbox items for each parent, were first converted into ordinal variables with values ranging from 1 to 6. Two composite variables were then computed: parental_education_mean, the arithmetic mean of maternal and paternal education levels (continuous).

Romantic partner status: Information about romantic relationships was recoded into two variables: (1) a binary variable has_partner (1 = has partner, 0 = does not have partner); and (2) a three-level psychosocial variable relational_status, reflecting relationship interest and status (0 = No partner and not interested, 1 = No partner but desires one, 2 = Currently has or has recently had a partner).

Data were screened for outliers and missingness using SPSS Statistics version 27 [26]. Cases with missing data were excluded listwise per analysis, which resulted in slight variations in the sample size (N) reported. All procedures were pre-defined in the statistical analysis plan. The overall missing data rate was low (<5%). Cases with missing values in variables required for a given analysis were excluded listwise. No imputation techniques were applied, as the missingness was deemed random and minimal.

##### Data Processing

Statistical analyses have been done using SPSS Statistics version 27 [26]. Normality was assessed using the Shapiro–Wilk test. Due to significant deviations (*p* < 0.05) for PHQ-9 and psychological predictors, non-parametric methods were prioritized where appropriate (Spearman’s correlation for PHQ-9). Descriptive statistics were computed. Group differences in PHQ-9 scores were evaluated using independent samples *t*-tests and one-way ANOVAs (with Tukey’s HSD post-hoc tests). Pearson correlations were calculated for continuous variables and visualized via heatmap. Multiple linear regression (for continuous PHQ-9 scores) and binary logistic regression (for PHQ-9 ≥ 10) were performed. Model assumptions were checked. We tested for multicollinearity using the variance inflation factor (VIF). All independent variables in the regression models had VIF values below 2, which indicates no significant multicollinearity. Odds ratios (ORs) with 95% confidence intervals (CI) were reported. Significance was set at α = 0.05 (two-tailed).

#### 2.1.4. Ethical Considerations

The Declaration of Helsinki’s ethical guidelines were followed in the study protocol. The Ethics Committee of the Galaţi College of Physicians provided their ethical approval (1165/29 November 2024). Every participant gave their informed consent electronically. Secure storage and unique identifiers were used to guarantee data confidentiality and anonymity. Because the systematic review component used previously published, non-identifiable data, it was considered exempt.

### 2.2. Systematic Review and Meta-Analysis

A systematic review of the literature was conducted following PRISMA guidelines to identify studies reporting depression prevalence among Romanian medical and dentistry students. Initially, we restricted inclusion to peer-reviewed, full-text, English- or Romanian-language studies reporting prevalence data on depression among Romanian medical or dental students. However, due to the extreme scarcity of eligible peer-reviewed studies (N = 2), we expanded our inclusion criteria to also consider: (1) studies published in conference proceedings or academic reports; (2) studies where only abstracts or partial data were available; and (3) non–peer-reviewed publications with extractable prevalence data and clearly described methods. This decision was made to enable a more meaningful synthesis of national evidence. We acknowledge that this expanded approach may introduce risk of bias related to quality assurance, selective reporting, and data completeness. These concerns are addressed in the Section 4 and factored into our interpretation of the pooled results.

#### 2.2.1. Search Strategy and Study Eligibility

Two authors (M.M. and P.C.C.) independently identified cross-sectional and longitudinal studies published before 12 April 2025 that report on depression prevalence or symptoms in Romanian medicine, dentistry, or nursing students. We systematically searched PubMed and Web of Science. Guided by the Preferred Reporting Items for Systematic Reviews and Meta-analyses (PRISMA) [27], reference lists of identified articles were screened, and study investigators were contacted if necessary. The search strategy combined terms related to depression prevalence/symptoms (including “Patient Health Questionnaire-9” OR “Beck Depression Index”) with terms for Romanian students, without language restriction (full details in Appendix A). References were managed using Rayyan (https://www.rayyan.ai/) [28]. After duplicate removal, titles and abstracts were screened; full texts were obtained for potentially eligible abstracts. Each full text was assessed independently for final inclusion. Disagreements were resolved by consensus. Peer-reviewed studies reporting depression prevalence in Romanian medical or dentistry students were included. Due to the small number (N = 2) that met the initial strict criteria, the search was expanded to include relevant articles that were not peer-reviewed or for which only abstracts were accessible, which yielded 4 additional studies suitable for prevalence estimation. The studies did not need to have depression as their primary outcome.

#### 2.2.2. Data Extraction and Quality Assessment

Two authors (M.C.P. and R.R.) independently extracted data using a standardized form: (1) study information (location, survey years, design, sample size, response rate, institutions); (2) participant characteristics (mean age, % women, specialty); (3) outcomes (depression prevalence, severity classification). Methodological quality was assessed using adapted criteria for prevalence studies from Hoy et al. (2012) [29] (Appendix B) to evaluate study design (longitudinal = strong, cross-sectional = weak), sample size (≥200 = strong), ascertainment measure quality (sensitivity/specificity > 75% = strong), sample representativeness (≥2 institutions = strong), and reporting of descriptive data. Disagreements were resolved by consensus.

#### 2.2.3. Statistical Analysis (Meta-Analysis)

All statistical analyses of the systematic review were done using RevMan version 5.4 [30]. A summary of depression prevalence, as reported in the included studies, was the primary goal. A forest plot was generated to visualize prevalence rates and confidence intervals. Heterogeneity was assessed using the I-squared statistic and the chi-square test. Due to significant heterogeneity, the pooled prevalence estimate was calculated using a random-effects model. A Z-test determined the statistical significance of the pooled prevalence.

To explore potential sources of heterogeneity, subgroup analyses were performed by stratifying studies based on the depression assessment instrument that was used (Beck Depression Inventory (BDI), Patient Health Questionnaire-9 (PHQ-9), Depression Anxiety Stress Scales-21 Items (DASS-21), Zung Self-Rating Depression Scale (ZSDS)).

Sensitivity analyses were also conducted to assess the robustness of the pooled prevalence estimate. These included (1) restricting the analysis to only peer-reviewed studies; and (2) restricting the analysis to only studies classified as having ‘Strong’ methodological quality based on Appendix B.

Both subgroup analyses and sensitivity analyses were performed using Python version 3.11.9 with pandas, numpy, and scipy libraries, with a random-effects model (DerSimonian-Laird method) being implemented to account for anticipated and observed heterogeneity. Heterogeneity was assessed using Cochran’s Q statistic and the I^2^ statistic, with I^2^ values of 0% indicating no heterogeneity, 25–50% low heterogeneity, 50–75% moderate to substantial heterogeneity, and >75% considerable heterogeneity.

## 3. Results

### 3.1. Local Cross-Sectional Study Results

#### 3.1.1. Sociodemographic Characteristics

A total of 495 undergraduate students from medical and nursing study programs, who were enrolled during the 2024–2025 academic year and were in their first three years of study, made up the study sample. The participants’ mean age was 25.14 years (SD = 9.67), with a median of 20.00 years (range = 18–54 years). Women made up 77.8% of the sample. General medicine accounted for the largest percentage of students (73.4%), followed by general nursing (24.0%). First-year students constituted the largest group (42.9%). Most participants originated from an urban residential environment (82.5%). The mean parental education level score was 2.97 (SD = 1.21). Approximately two-thirds reported having siblings (67.3%). A majority reported having a partner (63.9%) and engaging in hobbies (67.0%). Over half (52.2%) reported multiple sources of social support, while 8.5% reported none (Table 1).

#### 3.1.2. Prevalence and Severity of Depressive Symptoms (PHQ-9)

The average PHQ-9 score for the entire sample, which ranges from 0 to 27, was 8.43 (SD = 5.61, Median = 7.00), which suggests mild depressive symptomatology overall. Clinical cut-offs showed that 28.7% of respondents had minimal symptoms (0–4), 37.3% had mild symptoms (5–9), 19.6% had moderate symptoms (10–14), 9.3% had moderately severe symptoms (15–19), and 5.1% had severe symptoms (20–27). A total of 153 students, or 34.0% of the sample under analysis (N = 450 valid scores), had clinically significant depression (PHQ-9 score ≥ 10) (Table 2).

The significant proportion of students presented with mild to moderate depressive symptoms, which highlights the potential subclinical burden within this population.

**Figure 1:**
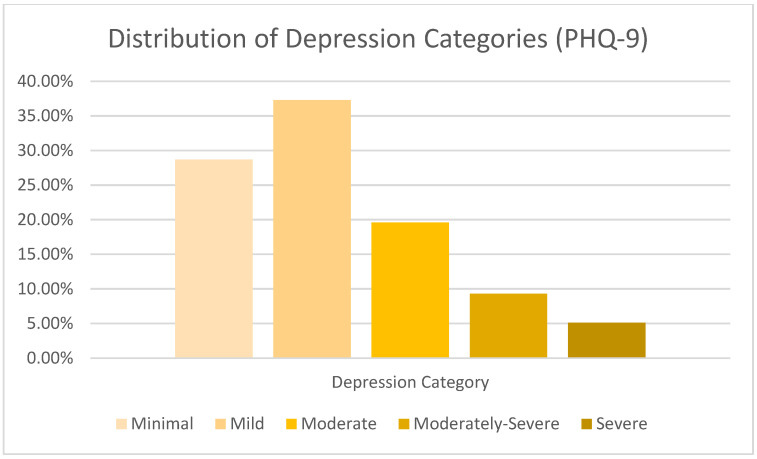
Distribution of depression severity categories (N = 450).

Moreover, a high percentage of students exhibited moderately severe to severe depression, which underscores the importance of accessible mental health resources and early intervention strategies (Figure 1).

#### 3.1.3. Correlates and Predictors of Depression in the Galați Sample

##### Group Differences in Depression Scores

**Figure 2:**
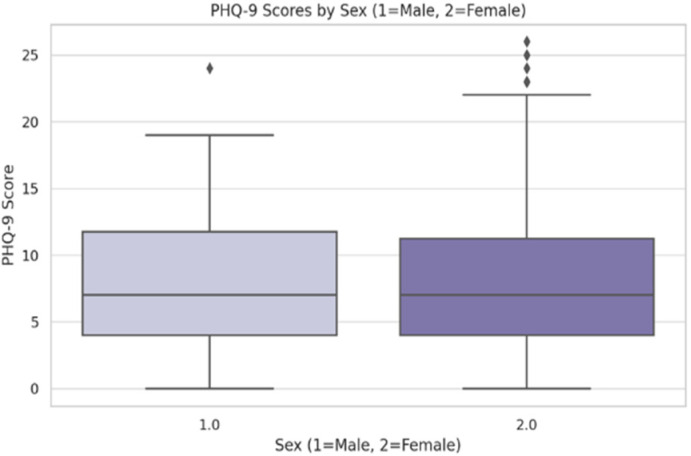
PHQ-9 scores by gender (N = 450). Error bars represent ±1 SD. The symbol represents outliers.

**Figure 3:**
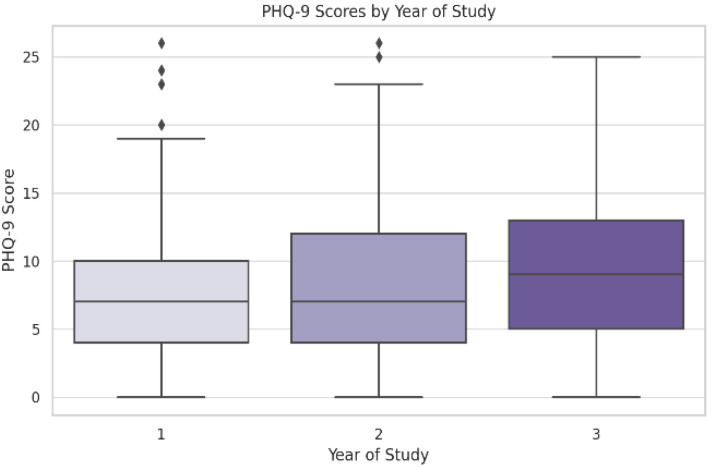
PHQ-9 scores by year of study. Error bars represent ±1 SD. The symbol represents outliers.

Inferential statistical analyses were used to examine the relationships between the PHQ-9 scores and various factors. Female students reported significantly higher depression scores than male students (M = 9.15 vs. M = 7.79, t (280.125) = −2.163, *p* = 0.031 (Figure 2 and Figure 3)).

**Figure 4:**
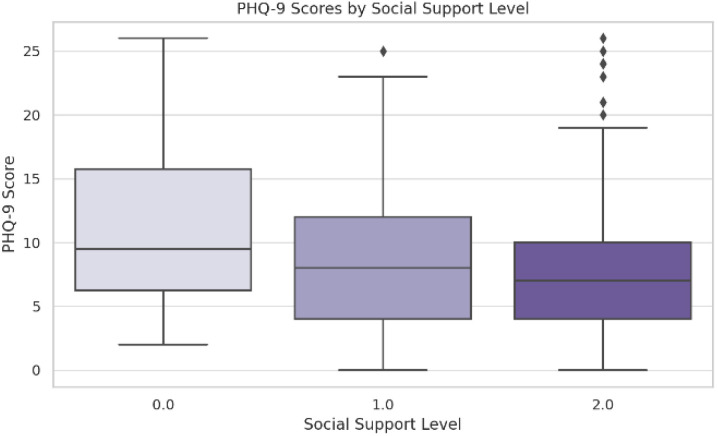
PHQ-9 scores by social support level. The symbol represents outliers.

The third-year students had significantly higher scores than the first-year students ((M = 9.99 vs. M = 8.09, Tukey HSD *p* = 0.038), ANOVA (F (2, 447) = 3.189, *p* = 0.042) (Table 3)). No significant differences between study programs were found (F (2, 447) = 0.802, *p* = 0.524). Lower perceived social support was significantly associated with higher depression scores (F (2, 447) = 3.929, *p* = 0.020), with those reporting no support scoring the highest (M = 11.61) compared to those with multiple sources (M = 8.20, Tukey HSD *p* = 0.018) (Figure 4).

**Figure 5:**
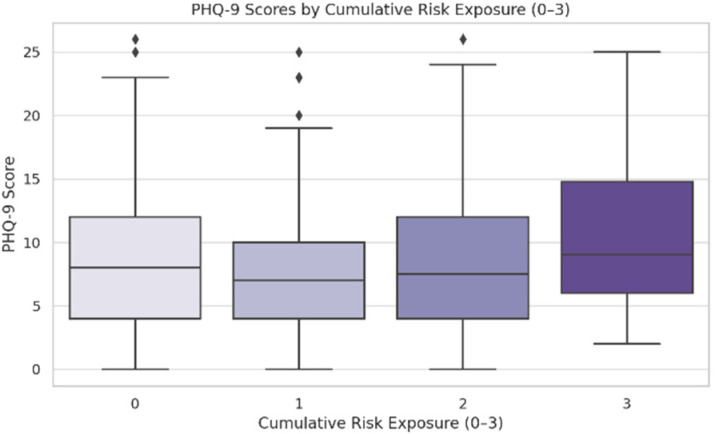
Scores by cumulative exposure to risk behaviors. The symbol represents outliers.

Higher depression scores were also positively correlated with cumulative exposure to risk behaviors (F (3, 446) = 2.68, *p* = 0.031), especially for those exposed to three or more risks as opposed to none (M = 11.03 vs. M = 9.50, Tukey HSD *p* = 0.031) (Figure 5). The PHQ-9 scores did not significantly correlate with having a partner (*p* = 0.66) or a hobby (*p* = 0.166).

##### Correlation and Regression Analyses

The PHQ-9 scores showed a moderately positive correlation with neuroticism (r = 0.34, *p* < 0.001) and a moderately negative correlation with conscientiousness (r = −0.31, *p* < 0.001), according to Pearson correlations. Additionally, there was a significant positive correlation with procrastination (r = 0.27, *p* < 0.001). Openness (r = −0.042, *p* = 0.376) and agreeableness (r = −0.083, *p* = 0.080) did not significantly correlate (*p* > 0.05); however, age showed a significant negative correlation (r = −0.172, *p* < 0.001).

Higher neuroticism (β = 1.49, *p* < 0.001) and lower conscientiousness (β = −1.17, *p* < 0.001) were found to be significant predictors of higher PHQ-9 scores by multiple linear regression analysis (excluding participants <18). Participants under the age of 18 were excluded from the regression analyses to ensure both ethical and analytical consistency. According to Romanian legislation and standard research ethics, individuals under 18 are considered minors and require additional protections, including parental or guardian consent. Including them could have introduced legal and procedural complexities, as well as ethical concerns. Moreover, minors may differ substantially from adults in terms of their cognitive, emotional, and psychological development, which could potentially affect how they experience and report depressive symptoms. Their inclusion could therefore introduce heterogeneity into the regression models and thus undermine the comparability and reliability of the results. Excluding this subgroup allowed us to maintain a more homogeneous and ethically sound analytical sample, in line with best practices for research involving adult university students.

Procrastination was also a significant positive predictor (β = 0.064, *p* = 0.027). Neither extroversion (β = −0.41, *p* = 0.146), social support (β = −0.81, *p* = 0.103), nor age (β = −0.04, *p* = 0.296) reached statistical significance in this model (Table 3).

Binary logistic regression predicting clinically significant depression (PHQ-9 ≥ 10) found that higher neuroticism significantly increased the odds (OR = 1.87, *p* < 0.001), as did higher procrastination scores (OR = 1.04, *p* = 0.003). Conversely, higher extroversion (OR = 0.77, *p* = 0.027) and older age (OR = 0.94, *p* = 0.006) were significant protective factors. Conscientiousness was not a significant predictor (OR = 1.05, *p* = 0.790) in this model (Table 4).

### 3.2. Systematic Review and Meta-Analysis Results

#### 3.2.1. Study Selection and Characteristics

**Figure 6:**
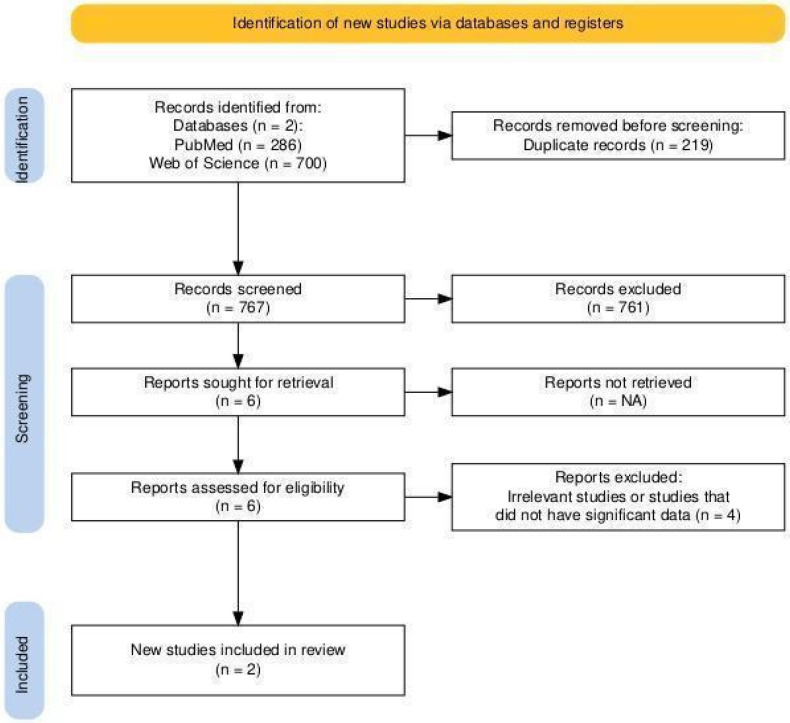
PRISMA flow diagram of literature search.

The systematic review followed the Prisma 2020 checklist (Supplementary Materials). A total of 986 articles from two databases were found using a systematic approach (Appendix A). Six articles were found to be initially eligible after duplicates were eliminated and their titles and abstracts were screened. Two peer-reviewed articles were included after the full-text review. Six studies in all were included in the final synthesis after a further search for pertinent non-peer-reviewed articles or abstracts turned up four more studies with prevalence data (Figure 6).

The six studies included 1546 students in total, from five different Romanian cities. Two studies were carried out in Timișoara, one in Iași, two in Cluj-Napoca (one of which was a joint study with Oradea), and two in Bucharest. The participants’ ages ranged from 19.5 to 25 years old on average. The study periods ranged from 2011 to 2024. The individual studies’ reported rates of depression varied widely, ranging from 14.2 to 64.2% (Table 5).

#### 3.2.2. Analysis of Prevalence

**Figure 7:**
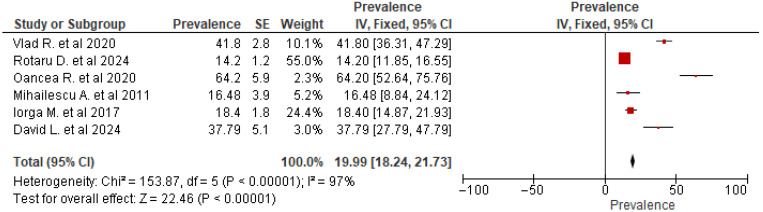
Forest plot for prevalence with pooled prevalence [16,17,18,20,31,32].

All statistical analysis was done in RevMan version 5.4 [30]. Due to the high variability in the reported prevalence across the included studies (14.2 to 64.2%), significant heterogeneity was confirmed (χ^2^ = 153.87, *p* < 0.00001, I^2^ = 87%). Therefore, a random-effects model was used to estimate the pooled prevalence. The pooled prevalence of depression among Romanian medical and dentistry students across these studies was 19.99% (95% confidence interval (CI) 18.24–21.73%). The pooled prevalence is unlikely to be the result of pure chance, as evidenced by the highly statistically significant overall effect size (Z = 22.46, *p* < 0.0001) (Figure 7).

The systematic review component also showed that gender, financial hardship, and academic stress were frequently mentioned as influencing depression in these Romanian studies.

#### 3.2.3. Subgroup Analysis by Assessment Instrument

To explore potential sources of the high overall heterogeneity, subgroup analyses were conducted based on the depression assessment instrument used in each study. The results are presented in Table 6.

As shown in Table 6, the pooled prevalence rates varied considerably across different instruments. The single study using PHQ-9 reported a prevalence of 63.97%, while the studies using the DASS-21 and ZSDS reported lower prevalences of 14.25 and 16.85%, respectively. The subgroup of studies using the BDI (N = 3) yielded a pooled prevalence of 32.82% (95% CI: 20.38–48.25), but still exhibited very high heterogeneity (I^2^ = 88.20%). These findings suggest that the choice of assessment instrument is a significant contributor to the observed overall heterogeneity, reflecting potential differences in diagnostic thresholds or symptom capture across tools.

#### 3.2.4. Sensitivity Analyses

Sensitivity analyses were performed to assess the robustness of the pooled prevalence estimate and to further explore sources of heterogeneity. The results are presented in Table 7.

When restricting the meta-analysis to only the two peer-reviewed studies, the pooled prevalence was 50.80% (95% CI: 26.63–74.61), with an I^2^ of 90.17%. Similarly, when including only studies classified as having ‘Strong’ methodological quality, the pooled prevalence was 26.20% (95% CI: 7.40–61.18), with an I^2^ of 99.05%. In both sensitivity analyses, the high level of heterogeneity persisted, which indicates that neither peer-review status nor the current ‘Strong’ quality classification adequately explained the substantial variability across studies.

## 4. Discussion

Numerous biological, psychological, and sociocultural factors contribute to the complexity of depression [33,34]. Numerous underlying mechanisms are suggested by neurobiological research, such as structural brain changes linked to emotional regulation, dysfunctions in neural networks, and changes in neurotransmitter systems (serotonin, dopamine, and norepinephrine) [35,36]. Significant contributions are also made by genetic predispositions, with the prevalence of particular variations varying among populations [37]. Furthermore, the prevalence, expression, resilience, and coping mechanisms of people with depression are significantly influenced by socio-cultural and environmental factors, including stress exposure, social support, cultural beliefs, and socioeconomic status [38,39]. Therefore, an integrative approach that takes into account the interaction of biological, genetic, and environmental factors is necessary to achieve a thorough understanding of depression.

Integrating a meta-analytic synthesis with our original data strengthens the relevance and interpretation of our findings. The systematic review allows us to position the Galați data against a pooled national prevalence, while also highlighting methodological inconsistencies in previous studies (e.g., variation in instruments, small samples). This juxtaposition reinforces the need for standardized research practices and situates our study as a model for both design and interpretation within the Romanian context. The thematic overlap, particularly around gender, academic pressure, and psychological traits, further confirms the validity of our findings.

### 4.1. Local Sample Results

Examining depression in this particular group is especially relevant when taking these factors, as well as the increased susceptibility of medical students, into account [9,10,11]. Untreated depression in medical students can have serious repercussions for the healthcare system, possibly affecting patient safety and effectiveness. Research indicates a correlation between physician depression and increased medical errors [1,38], highlighting the broader systemic implications. While our sample included both medical and nursing students, we recognize that the two tracks involve distinct educational demands and clinical exposure. Although faculty affiliation was accounted for as a covariate, future studies should consider stratified analyses or separate sampling to more precisely capture profession-specific mental health risk profiles.

While this study primarily focuses on Romanian medical students, its implications extend beyond the national context. Depression among medical students is a global concern, yet evidence from Eastern Europe remains underrepresented in the international literature. By providing both a detailed local investigation and the first systematic review and meta-analysis of Romanian studies, our research helps fill this geographic gap and allows for cross-national comparisons. Furthermore, by identifying procrastination and personality traits (particularly neuroticism and low conscientiousness) as significant correlates of depressive symptoms, this study contributes novel insights to international research efforts focused on psychological predictors. These findings may inform global mental health strategies, especially in medical education systems facing similar structural and cultural stressors.

Compared to the existing international research, this study adds new regional evidence to the global understanding of medical student mental health. While international meta-analyses report an average depression prevalence between 25 and 33%, our local finding of 34% in Galați is consistent with this trend and underscores the urgency of giving attention to underserved regions. Furthermore, by incorporating psychological correlates like procrastination and Big Five personality traits, our study extends international findings that often focus primarily on demographic and academic stress factors.

### 4.2. Meta-Analytic Findings

It is important to note that, to our knowledge, this represents the first attempt to conduct a systematic review of depression among medical students in Romania. We recognize that the inclusion of non–peer-reviewed studies and conference abstracts in the meta-analysis may reduce the methodological rigor of this study and introduce additional risk of bias. This approach was employed only after our initial search yielded an insufficient number of peer-reviewed articles, and all included studies were subjected to independent quality appraisal. Nonetheless, the pooled prevalence estimate should be interpreted cautiously, with full awareness of the heterogeneity and limitations inherent to the inclusion of grey literature. After loosening our inclusion criteria, we were able to obtain a pooled prevalence of depression, despite the fact that the initial inclusion criteria produced few articles for any meaningful analysis. However, the results should be carefully interpreted because there is a chance that bias will be introduced into the results due to the relaxation of the inclusion criteria. This meta-analytic synthesis of Romanian studies confirms that depression is a significant issue within the country’s medical and dental student populations, with a pooled prevalence of approximately 19.99% (95% CI (18.24, 21.73)). Despite being significant, this pooled estimate is less than the 34.0% rate of clinically significant depression (PHQ-9 ≥ 10) that was noted in our particular Galaţi sample. The high heterogeneity among the reviewed studies (I^2^ = 87%), the differences in the assessment tools used (BDI, PHQ-9, DASS, ZSDS), the sample characteristics (e.g., inclusion of nursing/dentistry vs. primarily medical), and the timing of the assessment in our study in relation to academic stressors are some of the possible causes of this discrepancy. Nonetheless, both the pooled estimate and our regional finding significantly exceed the prevalence rates that are typically reported for the general Romanian population [4,5], reinforcing the notion that medical training environments pose unique mental health challenges. Furthermore, to assess the robustness of our pooled prevalence estimate and explore sources of the high overall heterogeneity, sensitivity and subgroup analyses were conducted. A subgroup analysis by depression assessment instrument revealed considerable variation in the pooled prevalences. For instance, the single study using PHQ-9 reported a prevalence of 63.97%, whereas studies employing DASS-21 and ZSDS showed lower prevalences of 14.25 and 16.85%, respectively. Studies using the BDI yielded a pooled prevalence of 32.82%, although with persistently high heterogeneity. These findings suggest that the choice of assessment instrument significantly contributes to the observed heterogeneity, likely due to differences in diagnostic thresholds or symptom capture. Further sensitivity analyses were performed, first by including only peer-reviewed studies, which resulted in a pooled prevalence of 50.80%. Second, when including only studies classified as having ‘Strong’ methodological quality, the pooled prevalence was 26.20%. In both of these sensitivity analyses, the high level of heterogeneity persisted, indicating that neither peer-review status nor our ‘Strong’ quality classification adequately explained the substantial variability across studies. Comparatively, a 2023 meta-analysis investigating, among other factors, the prevalence of depression among students during the pandemic, which included 436,799 participants, revealed a student depression prevalence of 37% during that period [40]. Although this meta-analysis did not specifically target students from medical faculties, as in our study, the elevated depression rate that was observed is likely attributable to the pandemic context. Although our study did not include specific measures of COVID-19-related stress or disruption, it is plausible that the high prevalence of depressive symptoms observed in our sample (34.0%) reflects, in part, the lingering psychological effects of the pandemic. The academic year 2024–2025, during which data collection occurred, was relatively close to the post-pandemic recovery phase. During this period, students were still adjusting to the return of in-person instruction, navigating increased academic demands, and dealing with residual social disconnection or uncertainty about the future. These transitional stressors may have compounded the usual challenges of medical education. Studies conducted in other countries during the same post-pandemic period have similarly reported elevated mental health burdens among university students, even after lockdowns ended. Therefore, the elevated depression rates that we observed may be partially influenced by a “pandemic echo,” whereby psychological distress persists beyond the acute crisis phase. Future studies may benefit from including retrospective pandemic-related measures to better disentangle these effects.

In this context, our cross-sectional study contributes to the theoretical understanding of depression by empirically validating a set of psychological and personality-based predictors that are consistent with established models of emotional vulnerability. The significant role of procrastination supports prior findings that avoidance-based behaviors not only delay task completion but also exacerbate psychological strain and rumination. From the lens of cognitive-behavioral theory, such maladaptive coping strategies can fuel a cycle of perceived failure and negative self-appraisal, increasing the likelihood of depressive symptoms. Similarly, neuroticism emerged as a strong predictor, which aligns with the affective science literature and shows the centrality of this factor to emotional dysregulation and stress reactivity. Conscientiousness and extraversion, although more protective in nature, also showed expected inverse relationships with depressive symptoms. These patterns reflect the broader interplay between personality structure and coping mechanisms, which shape individuals’ responses to chronic academic and interpersonal stressors. By situating these findings within this theoretical landscape, this study contributes not only regionally relevant prevalence data, but also evidence for the broader applicability of personality-informed psychological models of student mental health.

Female students had higher depressive scores, which is in line with national [16,18,20] and international findings [8,13], even though there are studies that report no differences [41]. Although the results regarding the year of study can vary, the finding that third-year students reported higher levels of depression than first-year students is consistent with some of the literature that suggests accumulating stress over time [14]. Another potential explanation, possibly linked to local specificities, relates to the academic structure: the initial two years are preclinical, where students engage in learning patterns that are familiar from high school, primarily involving direct professor contact in lecture settings. The third year introduces clinical modules, necessitating practical engagement with patients in hospitals, emergency departments, and similar environments.

The results of the age analysis reveal a complex and seemingly inconsistent pattern across the different statistical approaches that were applied. We observed a significant negative correlation between age and continuous PHQ-9 scores (r = −0.172, *p* < 0.001), which suggests that older participants tend to report less severe depressive symptoms. However, in the linear regression model predicting continuous PHQ-9 scores, age was not a significant predictor (*p* = 0.296). This lack of significance might indicate that, while a general trend exists, the direct linear relationship between age and the continuous severity of depressive symptoms, when controlling for other factors, is not strong enough in our sample to reach statistical significance.

In contrast, the binary logistic regression analysis, which investigated the likelihood of meeting the criteria for clinical depression (PHQ-9 ≥ 10), revealed a significant protective effect of age (OR = 0.94, *p* = 0.006). This finding suggests that, for each additional year of age, the odds of being classified with clinical depression decrease by 6%. This discrepancy could be explained by age acting through a threshold or non-linear effect. For instance, as students mature, they may develop more effective coping mechanisms or experience a stabilization of stressors specific to the initial years of study, which thus influences the probability of reaching a clinically significant level of depression, even if finer variations in the continuous PHQ-9 score are not strongly associated with age in a linear model.

It is also important to note the unusually wide age range of our sample (18–54 years, median 20) compared to typical undergraduate medical student studies, which usually focus on much narrower age groups corresponding to the bachelor’s degree cycles. This heterogeneity in life stage could influence the interpretation of age-related findings. Older students in our sample might include individuals who interrupted their studies and resumed them or who pursued other educational paths before starting medicine, bringing with them different life experiences that could affect their vulnerability to depression. Therefore, this specific characteristic of our study population should be considered when interpreting the age-related results and may be mentioned as a potential limitation to the generalizability of our findings to medical student populations with more homogeneous age ranges.

A well-established finding in mental health research is the protective role of social support, which was found in our sample (*p* = 0.020) [4]. A substantial body of evidence consistently highlights a significant inverse relationship between the availability of robust social support networks and the prevalence or severity of depressive symptoms [42]. The presence of close confidants, individuals with whom one can openly share personal struggles and experiences, is frequently identified as a particularly potent protective factor against psychological distress, including depression [43]. Furthermore, the perceived adequacy and quality of these supportive relationships often matter more than the sheer number of social contacts, which suggests that deep, meaningful connections are central to this buffering effect [44]. However, it is pertinent to note that the relationship is not universally straightforward. Some research indicates nuances, suggesting that the type of support received or the specific population studied can moderate this association. For instance, while generally protective, poorly provided support or support from conflict-ridden relationships might not yield the expected mental health benefits, and certain studies in specific contexts have reported weaker or non-significant correlations, which warrants further investigation into these moderating factors [45].

Although social support showed a statistically significant negative correlation with depression scores in univariate analyses (*p* = 0.020), it did not remain a significant predictor in the multiple regression models. Several factors may account for this discrepancy. First, it is possible that the predictive variance attributed to social support overlaps with other variables in the model, particularly neuroticism, which is known to influence both perceived social support and depressive symptomatology. Second, the social support variable was ordinal and relatively coarse, which may have limited its sensitivity in a multivariate context. Third, the buffering effects of social support may operate more robustly as a moderator rather than a direct predictor, particularly under conditions of high stress or high neuroticism, which were not modeled in the current analysis. Finally, the loss of predictive significance may reflect the complexity of support quality and context, which a simple categorical variable may not fully capture. Future research may benefit from using more nuanced social support measures or testing interaction effects.

Higher depression scores are linked to cumulative risk behavior exposure (*p* = 0.031), which may indicate maladaptive coping or common underlying factors. Developmental trajectories towards depression are frequently influenced by the social contexts experienced during adolescence, with peer environments playing a particularly critical role [46]. A consistent body of research demonstrates a marked association between affiliation with peer groups that exhibit antisocial tendencies and an increased vulnerability to developing depressive symptoms during adolescence and young adulthood [47]. This includes exposure to peers who habitually disregard rules, engage in unlawful activities, have interactions with the criminal justice system, or normalize excessive alcohol consumption and substance use [48].

Such peer environments are theorized to heighten depression risk through multiple pathways, including the modeling of maladaptive behaviors, increased exposure to chronic stressors and negative life events, and potential social isolation from more prosocial peer networks. Nonetheless, the relationship between deviant peer affiliation and depression is not invariably deterministic, and some nuances warrant consideration. Research exploring reciprocal effects suggests that, while antisocial peers can increase depression risk (socialization effect), adolescents with pre-existing depressive symptoms might also be more likely to gravitate towards such peer groups (selection effect), which complicates causal interpretations [46,49]. Moreover, protective factors, such as strong family support or positive school engagement, can potentially buffer the adverse mental health consequences associated with exposure to antisocial peers, which indicates that the impact is not uniform across all individuals exposed to these risk factors [49].

The study sample’s psychological correlates are especially pertinent. The Five-Factor Model’s connections to emotional (dys)regulation and adaptive functioning [20] are strongly supported by the strong positive association between neuroticism and depression (r = 0.34, *p* < 0.001; significant predictor in regression models) and the negative association with conscientiousness (r = −0.31, *p* < 0.001; significant predictor in linear regression).

Neuroticism entails a predisposition towards anxiety, sadness, irritability, and heightened reactivity to stress [50,51]. Empirical evidence, particularly from meta-analyses and longitudinal studies, robustly supports the role of neuroticism as a vulnerability factor for depression. Meta-analyses indicate significant prospective associations between high neuroticism and increased risk for future depressive symptoms or episodes, even after adjusting for the baseline status [52,53]. Neuroticism predicts the initial onset of depression [54], and genetic and Mendelian randomization studies suggest a shared biological basis and a potential causal effect of neuroticism on depression [55,56].

Although neuroticism is a strong predictor of depression across diverse groups, certain socio-demographic factors can influence the expression of this link. For instance, stressors that are specific to particular life stages (e.g., adolescence, emerging adulthood) or social conditions (e.g., poverty, discrimination) might interact with neuroticism to elevate depression risk [57]. Some studies propose that the association could be more pronounced in women [58], although this requires further investigation, especially considering the higher overall prevalence of depression among women. In summary, neuroticism stands as a central vulnerability factor for depression, operating through an intricate network of cognitive, emotional, and behavioral mechanisms. Integrating the personality perspective into clinical practice and prevention initiatives is crucial for mitigating the burden of depression.

Conscientiousness frequently appears in the literature as being inversely correlated with depression [59,60,61]. As a core dimension of the Big Five model, conscientiousness reflects the tendency towards organization, diligence, goal-orientation, responsibility, and self-discipline [50,62]. It is worth noting that conscientiousness is multidimensional, encompassing facets like competence, orderliness, dutifulness, achievement-striving, and self-discipline [50]; thus, a detailed analysis of these facets is vital to obtain a nuanced understanding. The negative association between conscientiousness and depression is mediated via multiple pathways. Conscientious individuals tend to adopt healthier behaviors (e.g., physical activity, balanced nutrition, substance avoidance), which safeguard both physical and mental health [63,64]. They also employ more adaptive, problem-focused coping strategies (e.g., planning, problem-solving) over maladaptive ones (e.g., avoidance, denial) [65]. Their capacity to effectively set and pursue goals fosters feelings of self-efficacy and satisfaction [62,66]. Additionally, conscientiousness is linked to superior emotional regulation, including quicker recovery from negative affects. Self-regulation appears central to these interconnected mechanisms. The relationship is also likely bidirectional: low conscientiousness elevates depression risk, yet depression might, in turn, diminish conscientiousness levels (the “scar” hypothesis) [60,67].

Furthermore, extreme conscientiousness can manifest as maladaptive perfectionism (characterized by unrealistic standards and harsh self-criticism), a recognized risk factor for depression [68,69]. Inconsistent findings also exist within the literature, underscoring the importance of context and specific measurement approaches. In conclusion, while high conscientiousness generally offers protection, its relationship with depression is complex, influenced by specific facets, and potentially bidirectional, and may possess a ‘dark side’. A nuanced, facet-level approach is essential for future research and clinical application.

It is important to note that, although conscientiousness predicts the PHQ-9 score negatively and significantly in linear regression, the logistic model does not confirm this effect in the classification of clinical depression. This may reflect a linear relationship, but not one that is strong enough to discriminate between clinical and non-clinical cases.

One important finding from the logistic regression is that procrastination emerged as a significant predictor of clinically significant depression (OR = 1.04, *p* = 0.032), which may indicate the effect of avoidance, executive dysfunction, and the resulting academic stress on mood [40]. Although procrastination was a statistically significant predictor of clinically significant depression in the logistic regression model, the magnitude of this effect was small. This suggests that, while procrastination may contribute to depressive symptoms, its influence is likely subtle and accumulative rather than individually determinative.

The relationship between the Big Five personality trait of openness to experience and depression presents a complex picture within the existing literature. Some meta-analytic findings suggest a generally weak, albeit statistically significant, negative association, indicating that higher openness might be slightly protective against depressive symptoms [61]. Therefore, the finding in our study of a non-significant correlation (R = −0.042, *p* = 0.376) between openness, potentially measured using an instrument like the BFI-Short, and depression is not entirely anomalous and resonates with research that highlights the variability and often modest nature of this association [70]. Several studies, particularly those focusing on specific populations or controlling for multiple covariates, have reported no significant direct relationship between openness and depression scores. While neuroticism consistently emerges as a strong predictor, traits like openness often show weaker or non-significant associations with mood disorders in such research contexts. This may be due to the multifaceted nature of openness, which includes components such as intellectual curiosity, aesthetic sensitivity, and novelty-seeking. These facets may exert opposing effects, some potentially protective and others potentially linked to vulnerability through mechanisms like rumination, thereby weakening the overall correlation when measured as a single global construct [71]. Moreover, the relationship between personality traits and depression is often shaped by contextual moderators, such as stress, adversity, coping style, or cognitive flexibility, which may explain the variability in findings across samples [53]. In contrast, our results reinforce the protective roles of a younger age and higher extraversion against clinical depression, likely reflecting developmental resilience and greater reliance on social support networks.

An important limitation of our meta-analysis stems from the inclusion of non–peer-reviewed studies, abstracts with partial data, and gray literature. While this decision was necessary due to the limited number of peer-reviewed studies available on Romanian medical students, it introduces several potential sources of bias: (1) a lack of a standardized peer-review processes; (2) incomplete reporting of sample characteristics or methods; and (3) greater heterogeneity in study quality. Although we performed a basic methodological quality assessment and interpreted pooled prevalence estimates cautiously, we recommend that future national reviews be repeated with stricter criteria once more rigorous primary studies become available.

### 4.3. Cross-Comparison Between the Two

In contrast to the national estimates, our local data showed a substantially higher prevalence of affective symptoms. Despite the limitations brought about by study heterogeneity and the small number of eligible studies, the systematic review component supported regular reports of high rates of depression among Romanian medical students. These rates were frequently associated in the original studies with gender, financial difficulties, and academic stress, and these findings were consistent with the correlates identified in our Galaţi sample. The results of this study should be interpreted with several limitations in mind. First off, the study sample’s cross-sectional design makes it impossible to establish causal links between depression and its correlates; longitudinal research is necessary to understand the temporal dynamics. While the inclusion of non-peer-reviewed studies enabled us to estimate a national prevalence, it also introduces risk of methodological bias. Some included studies lacked full transparency in their sampling, measurement, or ethical oversight. Abstract-only studies may have inconsistent reporting, which makes it difficult to assess their data quality. Although we clearly labeled such inclusions and performed a sensitivity analysis, this limitation warrants caution in interpreting the pooled prevalence. Our study and the studies in the systematic review relied on self-report measures, which are susceptible to social desirability bias and recall bias, which may have affected the accuracy of symptom reporting and prevalence estimates. Thirdly, there were only six relevant studies in the Romanian context, and there was significant heterogeneity (I^2^ = 87%) among them with regard to prevalence rates, assessment instruments (PHQ-9, BDI, DASS-21, ZSDS), and student populations (medicine, dentistry, nursing). These factors limited the systematic review component. The pooled prevalence estimate (19.99%) should be interpreted with caution due to this heterogeneity, which exposes differences in national research methodology. Furthermore, differences in the depression screening instruments used across studies likely contributed to the observed heterogeneity. The Beck Depression Inventory (BDI) is a widely used self-report instrument with strong internal consistency (Cronbach’s alpha > 0.85) that is designed to assess the cognitive, affective, and somatic symptoms of depression. The Patient Health Questionnaire-9 (PHQ-9), based on DSM criteria, is brief and commonly used in clinical and non-clinical settings, with high sensitivity and specificity. The Depression Anxiety Stress Scales—21 (DASS-21) assesses a broader range of negative emotional states and may capture more general distress than clinical depression per se. The Zung Self-Rating Depression Scale (ZSDS) has been validated in diverse populations but is sometimes criticized for overlapping somatic items that may inflate scores in physically ill or stressed populations. These methodological differences, including differences in item content, response format, cut-off thresholds, and reference timeframes, can lead to substantial variation in reported prevalence rates and thus contributed to the high I^2^ value (87%) observed in our meta-analysis. A standardized approach to measurement in future studies could improve comparability and strengthen the reliability of combined estimates.

The variable methodological quality of the studies included in the meta-analysis, with three studies being rated as ‘weak’, may contribute to the substantial heterogeneity that was observed (I^2^ = 87%). This, along with the inclusion of non-peer-reviewed articles or abstracts (as noted in the Section 2), calls for cautious interpretation of the pooled prevalence estimate of 19.99% and underscores the need for future nationally representative studies that employ standardized and robust methodologies. Fourth, the majority of the studies in the review were from one large academic institution, and our sample was from one university in southeastern Romania. This may have limited the findings’ applicability to students in other regions or specializations within the country. Galați is a mid-sized city located in southeastern Romania, with a distinct sociocultural and economic profile compared to other major academic centers such as Bucharest, Cluj-Napoca, or Iași. It has traditionally been shaped by its industrial and maritime heritage, and while it hosts a growing academic environment, access to mental health infrastructure and extracurricular student opportunities may be more limited than in larger university hubs. Economic disparities between regions, differences in the student population composition (e.g., more first-generation university students), and varying levels of institutional mental health support could all influence the prevalence and expression of depression. Thus, while our findings reflect important trends in Romanian medical students, caution should be taken in generalizing the results to all academic settings in the country. Regional differences may play a meaningful role in student mental health and deserve further investigation through multicentric studies.

Lastly, the potential impact of unmeasured confounding variables warrants consideration. These include specific curriculum structures, the availability and utilization of university support services, and unrecorded personal life events. Furthermore, our cross-sectional design did not account for the significant variability in academic stress throughout the year, as the data collection was not specifically timed to capture experiences during high-stress periods like examinations versus more relaxed intervals. The aftereffects of the COVID-19 pandemic on specific cohorts also remain an unmeasured factor.

Both psychological traits and sociodemographic/contextual factors emerged as significant correlates or predictors of depression in the Galați sample. These convergent findings underscore the pressing need for proactive mental health support in Romanian medical education. Interventions should target the risk factors that have been identified, as well as social support networks, resilience and adaptive coping (especially regarding procrastination and neuroticism), and the incorporation of readily available psychological services into the demanding academic environment. To achieve this, strengthening the link between primary healthcare providers and existing community-based social services, potentially through the formalization of social prescribing pathways and the integration of “link workers” to facilitate referrals and patient engagement, is crucial [72].

For example, in the United Kingdom, the NHS has developed a national social prescribing framework that embeds link workers in general practice settings. These professionals assist patients, particularly those with mental health concerns, in accessing community resources, support groups, and wellbeing activities such as physical exercise, volunteering, or arts-based interventions. Preliminary evaluations have shown reductions in general practitioner visits, improvements in mental wellbeing, and increased social connectedness [73]. Similarly, Canadian universities have piloted campus-based link worker models that connect students to mental health resources both on- and off-campus. These programs often involve trained peer navigators or wellness coordinators who tailor their support based on students’ needs, and have demonstrated success in improving help-seeking behavior and reducing service fragmentation [74]. Adapting these models to Romanian universities could involve training psychology or social work graduates as liaison officers who are embedded in student health services and who would guide students toward both psychological services and non-clinical, community-based resources to enhance social connection and wellbeing.

This study offers a cross-sectional snapshot, yet it is a foundational component of a much broader research program. Our program’s ambition is to move beyond a singular focus on any one symptom, such as depression, and instead build a holistic, multidimensional understanding of the medical student experience in Romania. We are approaching this by weaving together distinct but complementary strands of research: the detailed analysis of psychosocial, behavioral, and lifestyle factors presented herein, alongside our parallel investigations into the neurobiological correlates of student distress [75].

Based on our findings, Romanian medical universities should consider implementing structured mental health screening at regular academic intervals, particularly around high-stress periods (e.g., exams, clinical transitions). Interventions that address neuroticism and procrastination, such as cognitive-behavioral skills training, time management workshops, and mindfulness-based stress reduction, may help reduce depression risk. Enhanced access to confidential counseling, faculty mental health training, and peer support systems are also vital steps toward cultivating a psychologically supportive academic culture.

## 5. Conclusions

Our study underscores the high burden of depression among Romanian medical and nursing students, a population that is essential to the future of healthcare and particularly vulnerable to mental health challenges. The prevalence of clinically significant depressive symptoms in our Galați sample (34.0%) notably exceeds both general population estimates and the pooled national prevalence (19.99%) identified through our systematic review and meta-analysis. This study highlights a high burden of depression among Romanian medical and nursing students, with 34% of the Galați sample meeting criteria for clinically significant symptoms. Depression was significantly associated with neuroticism, procrastination, female gender, lower social support, and an advanced year of study. Depression in this group is shaped by an interplay of psychological traits, especially high neuroticism and procrastination, and contextual vulnerabilities such as female gender, an advanced study year, low social support, and cumulative exposure to early adversity. These findings emphasize the need for structured mental health strategies in medical education that target both individual and systemic risk factors.

We advocate for the integration of accessible psychological services, early screening, and support mechanisms tailored to students’ developmental and academic realities. Strengthening connections between university services, primary care, and community-based resources, through approaches like social prescribing and link worker programs, could enhance the reach and effectiveness of interventions.

These findings underscore the urgent need for structured mental health support within Romanian medical education. Interventions should focus on early identification, access to psychological services, and programs that target maladaptive traits such as procrastination and emotional dysregulation.

Medical schools should consider implementing regular mental health screenings, resilience training, and integrated support systems that bridge academic and psychological care. Future research should adopt longitudinal and multicentric designs to explore causal pathways and inform scalable interventions.

This cross-sectional analysis forms part of a broader research agenda aimed at developing a comprehensive, multidimensional profile of student wellbeing in Romanian medical education. Future directions include a multicentric, longitudinal study to inform scalable, evidence-based interventions that are responsive to the evolving needs of Romania’s future healthcare workforce. A longitudinal and multicenter design would also allow for the exploration of causal relationships between psychological traits, contextual stressors, and mental health outcomes, the lack of which is an important limitation of the present cross-sectional approach. Furthermore, repeated assessments across semesters and academic years would enable the detection of seasonal or cyclical fluctuations in depression rates, such as those associated with examination periods, holidays, or clinical transitions. Capturing these temporal dynamics would provide a more nuanced and actionable understanding of mental health risks in medical education.

## Figures and Tables

**Table 1:** Sociodemographic characteristics of the sample (N = 495).

Characteristic	Category/Statistic	N	%	95% CI
**Age (years)**	Mean (SD)	495	25.14 (9.67) *	(24.29–25.99)
	Median (Range)		20.00 (18–54)	
**Gender**	Male	110	22.2	(24.29–25.99)
	Female	385	77.8	(73.7–81.4)
**Year of Study**	1	213	42.9	(38.5–47.4)
	2	113	22.8	(19.0–27.1)
	3	170	34.3	(30.0–38.8)
**Faculty**	1 (Medicine)	364	73.4	(69.2–77.2)
	2 (Nursing)	119	24.0	(20.0–28.4)
	3–6 (Other Health Related)	13	2.6	(1.4–4.7)
**Residence**	Urban	402	82.5	(78.8–85.7)
	Rural	12	2.5	(1.3–4.5)
	Suburban	73	15.0	(12.0–18.5)
**Parental Education**	Mean (SD)	314	2.97 (1.21)	(2.84–3.10)
	Median (Range)		3.00 (1–6)	
**Has Siblings**	No	162	32.7	(28.6–37.0)
	Yes	334	67.3	(63.0–71.4)
**Has Partner**	No	179	36.1	(31.9–40.5)
	Yes	317	63.9	(59.5–68.1)
**Has Hobby**	Yes	211	67.0	(61.6–72.0)
	No	104	33.0	(28.0–38.4)
**Social Support**	0 (None)	41	8.5	(6.2–11.5)
	1 (One source)	191	39.4	(34.8–44.1)
	2 (Multiple sources)	253	52.2	(47.4–57.0)

* Median (range) also presented for age due to skewed distribution.

**Table 2:** Distribution of depression severity categories based on PHQ-9 scores (N = 450).

Depression Category	Score Range	Count (N)	Percentage (%)	95% CI
Minimal	0–4	129	28.7	(24.6–33.1)
Mild	5–9	168	37.3	(32.8–41.9)
Moderate	10–14	88	19.6	(16.1–23.6)
Moderately Severe	15–19	42	9.3	(6.8–12.5)
Severe	20–27	23	5.1	(3.4–7.6)
**Total (Valid Scores)**		**450**	**100.00**	

**Table 3:** Multiple linear regression predicting PHQ-9 scores.

Predictor	Coef. (β)	Std. Err.	t	*p*-Value	VIF
(Intercept)	9.814	3.068	3.199	0.001	
Procrastination Score	0.064	0.025	2.219	0.027	1.387
Neuroticism	1.49	0.233	6.194	<0.001	1.110
Conscientiousness	−1.17	0.351	−3.22	<0.001	1.561
Extroversion	−0.41	0.271	−1.457	0.146	1.116
Social Support	−0.805	0.496	−1.633	0.103	1.051
Has Partner	0.152	0.657	0.23	0.818	1.119
Parental Educ. Mean	−0.279	0.258	−1.08	0.280	1.123
Age	−0.04	0.037	−1.047	0.296	1.391

**Table 4:** Binary logistic regression predicting clinically significant depression (PHQ-9 ≥ 10).

Predictor	Coef. (B)	Std. Err.	z	*p*-Value	OR (95% CI)	VIF
Procrastination Score	0.039	0.017	2.14	0.003	1.04 (1.00–1.07)	1.387
Neuroticism	0.477	0.095	4.95	<0.001	1.87 (1.55–2.27)	1.110
Conscientiousness	0.173	0.176	0.27	0.790	1.05 (0.74–1.48)	1.561
Extroversion	−0.220	0.099	−1.99	0.027	0.77 (0.60–0.99)	1.116
Social Support	−0.264	0.245	−1.08	0.280	0.77 (0.48–1.24)	1.051
Has Partner (1 = Yes)	−0.200	0.310	−0.64	0.520	0.82 (0.45–1.50)	1.119
Parental Educ. Mean	−0.193	0.125	−1.55	0.122	0.83 (0.65–1.05)	1.123
Age	−0.043	0.043	−2.72	0.006	0.94 (0.90–0.99)	1.391

Note: OR = odds ratio, CI = confidence interval.

**Table 5:** Characteristics of studies included in the systematic review and meta-analysis.

Author/Year	County/City	Sample Size	Specialty of Students	Female %	Age (Mean)	Depression N (%)	Instrument Used
Iorga M. et al., (2017) [18]	Iași	89	Nursing	91.0	25.52	16 (18.4%)	BDI
Vlad R. et al., (2020) [17]	Bucharest	315	Medical	83.0	22.86	136 (41.8%)	BDI
Oancea R. et al., (2020) [31]	Timișoara	67	NA	58.2	25.00	43 (64.2%)	PHQ-9
Rotaru D. et al., (2024) [20]	Cluj-Napoca Oradea	894	Dentistry	78.4	22.00	127 (14.2%)	DASS-21
Mihăilescu A. et al., (2011) [16]	Bucharest	91	Medical	82.6	19.50	15 (16.48%)	ZSDS
David L. et al., (2024) [32]	Cluj-Napoca	90	Nursing	91.1	21.00	34 (37.79%)	BDI

Note: NA = not available/applicable. BDI = Beck Depression Inventory; PHQ-9 = Patient Health Questionnaire-9; DASS-21 = Depression Anxiety Stress Scales—21 Items; ZSDS = Zung Self-Rating Depression Scale.

**Table 6:** Pooled prevalence of depression by assessment instrument (subgroup analysis).

Instrument	N Studies	Pooled Prevalence (%) (95% CI)	I^2^ (%)
BDI	3	32.82 (20.38–48.25)	88.2
PHQ-9	1	63.97 (51.88–74.51)	0
DASS-21	1	14.25 (12.10–16.69)	0
ZSDS	1	16.85 (10.48–25.97)	0

**Table 7:** Sensitivity analyses results.

Analysis Type	N Studies	Pooled Prevalence (%) (95% CI)	I^2^ (%)
Only Peer-Reviewed Studies	2	50.80 (26.63–74.61)	90.17
Only Studies with ‘Strong’ Quality	2	26.20 (7.40–61.18)	99.05

## Data Availability

The dataset generated during the current study is available in the Zenodo repository at https://doi.org/10.5281/zenodo.15671313. Access to the dataset is restricted to protect participant privacy. Data can be made available to researchers upon reasonable request submitted through the Zenodo access request system.

## Supplementary Materials

The following supporting information can be downloaded at: https://www.mdpi.com/article/10.3390/jcm14165853/s1, Prisma 2020 checklist. Ref. [76] is cited in the Supplementary Materials.

## Abbreviations

The following abbreviations are used in this manuscript:

ANOVA	Analysis of Variance
BDI	Beck Depression Inventory
BFI-S	Big Five Inventory—Short
CC BY	Creative Commons Attribution
CI	Confidence Interval
DASS-21	Depression Anxiety Stress Scales—21 Items
HSD	Honestly Significant Difference (Tukey’s)
I^2^	I-Squared Statistic (Heterogeneity)
M	Mean
NA	Not Available/Not Applicable
OR	Odds Ratio
PHQ-9	Patient Health Questionnaire-9
PRISMA	Preferred Reporting Items for Systematic Reviews and Meta-analyses
SD	Standard Deviation
SPSS	Statistical Package for the Social Sciences
ZSDS	Zung Self-Rating Depression Scale

## Appendix A

**Table A1:** Search strategy protocol.

Source	Search Strategy	Articles Retrieved
**PubMed**	((((((((Depression prevalence) OR (depression symptoms)) AND (medicine students)) OR (dentistry students)) AND (PHQ-9)) OR (Patient Health Questionnaire)) OR (BDI)) OR (Beck Depression Inventory)) AND (Romania) Filters: Free Full Text	286
**Web of Science**	((((((((ALL = (Depression prevalence)) OR ALL = (depression symptoms)) AND ALL = (medicine students)) OR ALL = (dentistry students)) AND ALL = (PHQ-9)) OR ALL = (Patient Health Questionnaire)) OR ALL = (BDI)) OR ALL = (Beck Depression Inventory)) AND ALL = (Romania) Filters: Open Access and Countries/Regions = Romania	700

## Appendix B

**Table A2:** Methodological quality assessment.

No	Author/Year	Study Design		Sample Size		Ascertainment of Depressive Symptoms Measure		Representativeness of the Sample		Descriptive Characteristics of Participants		Overall Methodological Quality	
		Auth 1	Auth 2	Auth 1	Auth 2	Auth 1	Auth 2	Auth 1	Auth 2	Auth 1	Auth 2	Auth 1	Auth 2
1	Iorga M. et al./2017 [18]	W	W	W	W	S	S	W	W	S	S	W	W
2	Vlad R. et al./2020 [17]	W	W	S	S	S	S	S	S	S	S	S	S
3	Oancea R. et al./2020 [31]	W	W	W	W	S	S	W	W	S	S	W	W
4	Rotaru D. et al./2024 [20]	S	S	S	S	S	S	S	S	S	S	S	S
5	Mihăilescu A. et al./2011 [16]	W	W	W	W	S	S	W	W	S	S	W	W
6	David L. et al./2024 [32]	W	W	W	W	S	S	W	W	S	S	W	W

W = weak; S = strong.

## References

[1] World Health Organization (2023). Depression. https://www.who.int/news-room/fact-sheets/detail/depression.

[2] Vos T., Lim S.S., Abbafati C., Abbas K.M., Abbasi M., Abbasifard M., Abbasi-Kangevari M., Abbastabar H., Abd-Allah F., Abdelalim A. (2020). Global burden of 369 diseases and injuries in 204 countries and territories, 1990–2019: A systematic analysis for the Global Burden of Disease Study 2019. Lancet.

[3] OECD, European Union (2022). Health at a Glance: Europe 2022: State of Health in the EU Cycle.

[4] Consiliul Economic și Social (CES) (2023). Sănătatea Mintală în România: Un Tablou Integrative.

[5] Patriche D., Filip I., Tănase C. (2015). Epidemiologia depresiei. Rev. Med. Rom..

[6] Balazs J., Miklósi M., Keresztény Á., Apter A., Bobes J., Brunner R., Corcoran P., Cosman D., Haring C., Kahn J.-P. (2012). P-259-Prevalence of adolescent depression in Europe. Eur. Psychiatry.

[7] Iovu M.B., Breaz M.A. (2019). The prevalence and burden of mental and substance use disorders in Romania: Findings from the Global Burden of Disease Study 2016. Psychiatr. Danub..

[8] Ibrahim A.K., Kelly S.J., Adams C.E., Glazebrook C. (2013). A systematic review of studies of depression prevalence in university students. J. Psychiatr. Res..

[9] Rotenstein L.S., Ramos M.A., Torre M., Segal J.B., Peluso M.J., Guille C., Sen S., Mata D.A. (2016). Prevalence of depression, depressive symptoms, and suicidal ideation among medical students: A systematic review and meta-analysis. JAMA.

[10] Mata D.A., Ramos M.A., Bansal N., Khan R., Guille C., Di Angelantonio E., Sen S. (2015). Prevalence of depression and depressive symptoms among resident physicians: A systematic review and meta-analysis. JAMA.

[11] Puthran R., Zhang M.W., Tam W.W., Ho R.C. (2016). Prevalence of depression amongst medical students: A meta-analysis. Med. Educ..

[12] Quek T.T.-C., Tam W.S., Tran B.X., Zhang M., Zhang Z., Ho C.S.-H., Ho R.C.-M. (2019). The global prevalence of anxiety among medical students: A meta-analysis. Int. J. Environ. Res. Public. Health.

[13] Dyrbye L.N., Thomas M.R., Shanafelt T.D. (2006). Systematic review of depression, anxiety, and other indicators of psychological distress among U.S. and Canadian medical students. Acad. Med..

[14] Yusoff M.S.B., Abdul Rahim A.F., Baba A.A., Ismail S.B., Mat Pa M.N., Esa A.R. (2013). The impact of medical education on psychological health of students: A cohort study. Psychol. Health Med..

[15] Dahlin M., Joneborg N., Runeson B. (2005). Stress and depression among medical students: A cross-sectional study. Med. Educ..

[16] Mihăilescu A., Matei V., Cioca I., Iamandescu I. (2011). Stresul perceput-predictor al anxietății și depresiei la un grup de studenți în primul an la medicină. Pract. Med..

[17] Vlad R. (2020). Depression and anxiety in Romanian medical students: Prevalence and associations with personality. Farmacia.

[18] Iorga M., Muraru D., Soponaru C., Petrariu F. (2017). Factors influencing the level of depression among freshman nursing students. Med. Surg. J..

[19] Sfeatcu R., Balgiu B.A., Parlătescu I. (2021). New psychometric evidences on the Dental Environment Stress questionnaire among Romanian students. J. Educ. Health Promot..

[20] Rotaru D.I., Chișnoiu R.M., Bolboacă S.D., Gileru E.A., Chișnoiu A.M., Delean A.G. (2024). Insights into self-evaluated stress, anxiety, and depression among dental students. Sci. Rep..

[21] Zugun-Eloae C., Iorga M., Gavrilescu I.M., Florea S.G., Chelaru A. (2016). Motivation, stress and satisfaction among medical students. Med. Surg. J..

[22] Stoet G. (2010). PsyToolkit: A software package for programming psychological experiments using Linux. Behav. Res. Methods.

[23] Kroenke K., Spitzer R.L., Williams J.B.W. (2001). The PHQ-9: Validity of a brief depression severity measure. J. Gen. Intern. Med..

[24] Lay C.H. (1986). At last, my research article on procrastination. J. Res. Pers..

[25] Gerlitz J.-Y., Schupp J. (2005). Zur Erhebung der Big-Five-basierten Persönlichkeitsmerkmale im SOEP: Dokumentation der Instrumentenentwicklung BFI-S auf Basis des SOEP-Pretests. DIW Res. Notes.

[26] IBM Corp (2020). IBM SPSS Statistics for Windows [Computer Software].

[27] Haddaway N.R., Page M.J., Pritchard C.C., McGuinness L.A. (2022). PRISMA2020: An R package and Shiny app for producing PRISMA 2020-compliant flow diagrams, with interactivity for optimised digital transparency and Open Synthesis. Campbell Syst. Rev..

[28] Ouzzani M., Hammady H., Fedorowicz Z., Elmagarmid A. (2016). Rayyan—A web and mobile app for systematic reviews. Syst. Rev..

[29] Hoy D., Brooks P., Woolf A., Blyth F., March L., Bain C., Baker P., Smith E., Buchbinder R. (2012). Assessing risk of bias in prevalence studies: Modification of an existing tool and evidence of interrater agreement. J. Clin. Epidemiol..

[30] The Cochrane Collaboration Review Manager (RevMan) [Internet]; 2020. https://Revman.cochrane.org.

[31] Oancea R., Timar B., Papava I., Cristina B.A., Ilie A.C., Dehelean L. (2020). Influence of depression and self-esteem on oral health-related quality of life in students. J. Int. Med. Res..

[32] David L., Ismaiel A., Faucambert P., Leucuta D.C., Popa S.F., Fadgyas S.M., Dumitrascu D.L. (2024). Mental Disorders, Social Media Addiction, and Academic Performance in Romanian Undergraduate Nursing Students. JCM.

[33] Dean J., Keshavan M. (2017). The neurobiology of depression: An integrated view. Asian J. Psychiatry.

[34] Otte C., Gold S.M., Penninx B.W., Pariante C.M., Etkin A., Fava M., Mohr D.C., Schatzberg A.F. (2016). Major Depressive Disorder. Nat Rev Dis Primers.

[35] Howard D.M., Adams M.J., Shirali M., Clarke T.-K., Marioni R.E., Davies G., Coleman J.R.I., Alloza C., Shen X., Barbu M.C. (2018). Genome-Wide Association Study of Depression Phenotypes in UK Biobank Identifies Variants in Excitatory Synaptic Pathways. Nat Commun.

[36] Kirmayer L.J., Gomez-Carrillo A., Veissière S. (2017). Culture and Depression in Global Mental Health: An Ecosocial Approach to the Phenomenology of Psychiatric Disorders. Soc. Sci. Med..

[37] Lorant V., Deliège D., Eaton W., Robert A., Philippot P., Ansseau M. (2003). Socioeconomic inequalities in depression: A meta-analysis. Am. J. Epidemiol..

[38] Fahrenkopf A.M., Sectish T.C., Barger L.K., Sharek P.J., Lewin D., Chiang V.W., Edwards S., Wiedermann B.L., Landrigan C.P. (2008). Rates of medication errors among depressed and burnt out residents: Prospective cohort study. BMJ.

[39] Ionescu C.G., Chendea A., Licu M. (2023). Is satisfaction with online learning related to depression, anxiety, and insomnia symptoms? A cross-sectional study on medical undergraduates in Romania. Eur. J. Investig. Health Psychol. Educ..

[40] Wang C., Wen W., Zhang H., Ni J., Jiang J., Cheng Y., Zhou M., Ye L., Feng Z., Ge Z. (2023). Anxiety, depression, and stress prevalence among college students during the COVID-19 pandemic: A systematic review and meta-analysis. J. Am. Coll. Health.

[41] Moreno-Agostino D., Wu Y.-T., Daskalopoulou C., Hasan M.T., Huisman M., Prina M. (2021). Global trends in the prevalence and incidence of depression: A systematic review and meta-analysis. J. Affect. Disord..

[42] Santini Z.I., Koyanagi A., Tyrovolas S., Mason C., Haro J.M. (2015). The association between social relationships and depression: A systematic review. J. Affect. Disord..

[43] Li G., Li Y., Lam A.I.F., Tang W., Seedat S., Barbui C., Papola D., Panter-Brick C., van der Waerden J., Bryant R. (2023). Understanding the protective effect of social support on depression symptomatology from a longitudinal network perspective. BMJ Ment Health.

[44] Wang J., Mann F., Lloyd-Evans B., Ma R., Johnson S. (2018). Associations between loneliness and perceived social support and outcomes of mental health problems: A systematic review. BMC Psychiatry.

[45] Hefner J., Eisenberg D. (2009). Social support and mental health among college students. Am. J. Orthopsychiatry.

[46] McLaughlin K.A., King K. (2015). Developmental trajectories of anxiety and depression in early adolescence. J. Abnorm. Child Psychol..

[47] Heerde J.A., Curtis A., Bailey J.A., Smith R., Hemphill S.A., Toumbourou J.W. (2019). Reciprocal associations between early adolescent antisocial behavior and depressive symptoms: A longitudinal study in Victoria, Australia and Washington State, United States. J. Crim. Justice.

[48] Reinke W.M., Eddy J.M., Dishion T.J., Reid J.B. (2012). Joint trajectories of symptoms of disruptive behavior problems and depressive symptoms during early adolescence and adjustment problems during emerging adulthood. J. Abnorm. Child Psychol..

[49] Brendgen M., Vitaro F., Bukowski W.M., Dionne G., Tremblay R.E., Boivin M. (2013). Can friends protect genetically vulnerable children from depression?. Dev. Psychopathol..

[50] Costa P.T. (1992). Revised NEO Personality Inventory (NEO PI-R) and NEP Five-Factor Inventory (NEO-FFI): Professional Manual.

[51] Widiger T.A., Oltmanns J.R. (2017). Neuroticism is a fundamental domain of personality with enormous public health implications. World Psychiatry.

[52] Hakulinen C., Elovainio M., Pulkki-Råback L., Virtanen M., Kivimäki M., Jokela M. (2015). Personality and depressive symptoms: Individual participant meta-analysis of 10 cohort studies. Depress Anxiety.

[53] Ormel J., Jeronimus B.F., Kotov R., Riese H., Bos E.H., Hankin B., Rosmalen J.G.M., Oldehinkel A.J. (2013). Neuroticism and common mental disorders: Meaning and utility of a complex relationship. Clin. Psychol. Rev..

[54] Kendler K.S., Kuhn J., Prescott C.A. (2004). The interrelationship of neuroticism, sex, and stressful life events in the prediction of episodes of major depression. Am. J. Psychiatry.

[55] Nagel M., Jansen P.R., Stringer S., Watanabe K., de Leeuw C.A., Bryois J., Savage J.E., Hammerschlag A.R., Skene N.G., Muñoz-Manchado A.B. (2018). Meta-analysis of genome-wide association studies for neuroticism in 449,484 individuals identifies novel genetic loci and pathways. Nat. Genet..

[56] Luciano M., Hagenaars S.P., Davies G., Hill W.D., Clarke T.-K., Shirali M., Harris S.E., Marioni R.E., Liewald D.C., Fawns-Ritchie C. (2018). Association analysis in over 329,000 individuals identifies 116 independent variants influencing neuroticism. Nat. Genet..

[57] Oldehinkel A.J., Hartman C.A., De Winter A.F., Veenstra R., Ormel J. (2004). Temperament profiles associated with internalizing and externalizing problems in preadolescence. Dev. Psychopathol..

[58] Morken I.S., Viddal K.R., von Soest T., Wichstrøm L. (2023). Explaining the female preponderance in adolescent depression: A four-wave cohort study. Res. Child Adolesc. Psychopathol..

[59] Packer J., Flack M. (2024). The role of self-esteem, depressive symptoms, extraversion, neuroticism and FOMO in problematic social media use: Exploring user profiles. Int. J. Ment. Health Addict..

[60] Johnson M.D., Galambos N.L., Krahn H.J. (2016). Vulnerability, scar, or reciprocal risk? Temporal ordering of self-esteem and depressive symptoms over 25 years. Longitud. Life Course Stud..

[61] Kotov R., Gamez W., Schmidt F., Watson D. (2010). Linking “big” personality traits to anxiety, depressive, and substance use disorders: A meta-analysis. Psychol. Bull..

[62] Roberts B.W., Walton K.E., Bogg T. (2005). Conscientiousness and health across the life course. Rev. Gen. Psychol..

[63] Bogg T., Roberts B.W. (2004). Conscientiousness and health-related behaviors: A meta-analysis of the leading behavioral contributors to mortality. Psychol. Bull..

[64] Joyner C., Rhodes R.E., Loprinzi P.D. (2018). The prospective association between the five factor personality model with health behaviors and health behavior clusters. Eur. J. Psychol..

[65] Connor-Smith J.K., Flachsbart C. (2007). Relations between personality and coping: A meta-analysis. J. Pers. Soc. Psychol..

[66] Hayat A.A., Kohoulat N., Amini M., Faghihi S.A.A. (2020). The predictive role of personality traits on academic performance of medical students: The mediating role of self-efficacy. Med. J. Islam. Repub. Iran.

[67] Klein D.N., Kotov R., Bufferd S.J. (2011). Personality and depression: Explanatory models and review of the evidence. Annu. Rev. Clin. Psychol..

[68] Smith M.M., Sherry S.B., Ray C., Hewitt P.L., Flett G.L. (2021). Is perfectionism a vulnerability factor for depressive symptoms, a complication of depressive symptoms, or both? a meta-analytic test of 67 longitudinal studies. Clin. Psychol. Rev..

[69] Li N., Zhang X., Zheng Y., Liu Q., Niu S., Qin Y., Zhang Y., Liu Y., Wang J. (2024). The impact of perfectionism on the incidence of major depression in Chinese medical freshmen: From a 1-year longitudinal study. Psychol. Res. Behav. Manag..

[70] DeYoung C.G., Quilty L.C., Peterson J.B. (2007). Between facets and domains: 10 aspects of the Big Five. J. Pers. Soc. Psychol..

[71] Fabretti R.R., Zanon C. (2024). Rumination is differentially related to openness and intellect. Pers. Individ. Dif..

[72] Surugiu R., Iancu M.A., Lăcătus A.M., Dogaru C.A., Stepan M.D., Eremia I.A., Neculau A.E., Dumitra G.G. (2023). Unveiling the Presence of Social Prescribing in Romania in the Context of Sustainable Healthcare—A Scoping Review. Sustainability.

[73] Aughterson H., Fancourt D., Chatterjee H., Burton A. (2024). Social prescribing for individuals with mental health problems: An ethnographic study exploring the mechanisms of action through which community groups support psychosocial well-being. Wellcome Open Res..

[74] Ecclestone A., Linden B., Rose J., Kullar K. (2025). Mobilizing health promotion through canada’s student mental health network: Concurrent, mixed methods process evaluation. JMIR Form. Res..

[75] Plesea-Condratovici C., Plesea-Condratovici A., Dinu C.A., Nicolcescu P., Robles-Rivera K., Weiser M., Mutica M., Burlea L.S., Ciubara A. (2025). The Cognitive and Behavioural Impact of Social Media and Gaming on Academic Performance in Medical and Nursing Students. BRAIN Broad Res. Artif. Intell. Neurosci..

[76] Page M.J., McKenzie J.E., Bossuyt P.M., Boutron I., Hoffmann T.C., Mulrow C.D., Shamseer L., Tetzlaff J.M., Akl E.A., Brennan S.E. (2021). The PRISMA 2020 statement: An updated guideline for reporting systematic reviews. BMJ.

